# Utility of Plasma GDF-15 for Diagnosis and Prognosis Assessment of ICU-Acquired Weakness in Mechanically Ventilated Patients: Prospective Observational Study

**DOI:** 10.1155/2020/3630568

**Published:** 2020-02-11

**Authors:** Yongpeng Xie, Suxia Liu, Hui Zheng, Lijuan Cao, Kexi Liu, Xiaomin Li

**Affiliations:** ^1^Department of Critical Care Medicine, Lianyungang Clinical College of Nanjing Medical University, The First People's Hospital of Lianyungang City, Lianyungang, China; ^2^Department of Emergency Medicine, Lianyungang Clinical College of Nanjing Medical University, The First People's Hospital of Lianyungang City, Lianyungang, China

## Abstract

**Objective:**

To identify the clinical correlations between plasma growth differentiation factor-15 (GDF-15), skeletal muscle function, and acute muscle wasting in ICU patients with mechanical ventilation. In addition, to investigate its diagnostic value for ICU-acquired weakness (ICU-AW) and its predictive value for 90-day survival in mechanically ventilated patients.

**Methods:**

95 patients with acute respiratory failure, who required mechanical ventilation therapy, were randomly selected among hospitalized patients from June 2017 to January 2019. The plasma GDF-15 level was detected by ELISA, the rectus femoris cross-sectional area (RFcsa) was measured by ultrasound, and the patient's muscle strength was assessed using the British Medical Research Council (MRC) muscle strength score on day 1, day 4, and day 7. Patients were divided into an ICU-AW group and a non-ICU-AW group according to their MRC-score on the 7th day. The differences in plasma GDF-15 level, MRC-score, and RFcsa between the two groups were compared on the 1st, 4th, and 7th day after being admitted to the ICU. Then, the correlations between plasma GDF-15 level, RFcsa loss, and MRC-score on day 7 were investigated. The receiver operating characteristic curve (ROC) was used to analyze the plasma GDF-15 level, RFcsa loss, and % decrease in RFcsa on the 7th day to the diagnosis of ICU-AW in mechanically ventilated patients. Moreover, the predictive value of GDF-15 on the 90-day survival status of patients was assessed using patient survival curves.

**Results:**

Based on whether the 7th day MRC-score was <48, 50 cases were included in the ICU-AW group and 45 cases in the non-ICU-AW group. The length of mechanical ventilation, ICU length of stay, and hospital length of stay were significantly longer in the ICU-AW group than in the non-ICU-AW group (all *P* < 0.05), while the other baseline indicators were not statistically significant between the two groups. As the treatment time increased, the plasma GDF-15 level was significantly increased, the ICU-AW group demonstrated a significant decreasing trend in the MRC-score and RFcsa, while no significant changes were found in the non-ICU-AW group. In the ICU-AW group, the plasma GDF-15 level was significantly higher than that in the non-ICU-AW group, while the RFcsa and the MRC-score were significantly lower than those in the non-ICU-AW group (GDF-15 (pg/ml): 2542.44 ± 629.38 vs. 1542.86 ± 502.86; RFcsa (cm^2^): 2.04 ± 0.64 vs. 2.34 ± 0.61; MRC-score: 41.22 ± 3.42 vs. 51.42 ± 2.72, all *P* < 0.05), while the other baseline indicators were not statistically significant between the two groups. As the treatment time increased, the plasma GDF-15 level was significantly increased, the ICU-AW group demonstrated a significant decreasing trend in the MRC-score and RFcsa, while no significant changes were found in the non-ICU-AW group. In the ICU-AW group, the plasma GDF-15 level was significantly higher than that in the non-ICU-AW group, while the RFcsa and the MRC-score were significantly lower than those in the non-ICU-AW group (GDF-15 (pg/ml): 2542.44 ± 629.38 vs. 1542.86 ± 502.86; RFcsa (cm^2^): 2.04 ± 0.64 vs. 2.34 ± 0.61; MRC-score: 41.22 ± 3.42 vs. 51.42 ± 2.72, all *r* = −0.60), while it was significantly positively correlated with the RFcsa loss (*r* = −0.60), while it was significantly positively correlated with the RFcsa loss (*r* = −0.60), while it was significantly positively correlated with the RFcsa loss (*r* = −0.60), while it was significantly positively correlated with the RFcsa loss (*P* < 0.05), while the other baseline indicators were not statistically significant between the two groups. As the treatment time increased, the plasma GDF-15 level was significantly increased, the ICU-AW group demonstrated a significant decreasing trend in the MRC-score and RFcsa, while no significant changes were found in the non-ICU-AW group. In the ICU-AW group, the plasma GDF-15 level was significantly higher than that in the non-ICU-AW group, while the RFcsa and the MRC-score were significantly lower than those in the non-ICU-AW group (GDF-15 (pg/ml): 2542.44 ± 629.38 vs. 1542.86 ± 502.86; RFcsa (cm^2^): 2.04 ± 0.64 vs. 2.34 ± 0.61; MRC-score: 41.22 ± 3.42 vs. 51.42 ± 2.72, all *P* < 0.05), while the other baseline indicators were not statistically significant between the two groups. As the treatment time increased, the plasma GDF-15 level was significantly increased, the ICU-AW group demonstrated a significant decreasing trend in the MRC-score and RFcsa, while no significant changes were found in the non-ICU-AW group. In the ICU-AW group, the plasma GDF-15 level was significantly higher than that in the non-ICU-AW group, while the RFcsa and the MRC-score were significantly lower than those in the non-ICU-AW group (GDF-15 (pg/ml): 2542.44 ± 629.38 vs. 1542.86 ± 502.86; RFcsa (cm^2^): 2.04 ± 0.64 vs. 2.34 ± 0.61; MRC-score: 41.22 ± 3.42 vs. 51.42 ± 2.72, all *P* < 0.05), while the other baseline indicators were not statistically significant between the two groups. As the treatment time increased, the plasma GDF-15 level was significantly increased, the ICU-AW group demonstrated a significant decreasing trend in the MRC-score and RFcsa, while no significant changes were found in the non-ICU-AW group. In the ICU-AW group, the plasma GDF-15 level was significantly higher than that in the non-ICU-AW group, while the RFcsa and the MRC-score were significantly lower than those in the non-ICU-AW group (GDF-15 (pg/ml): 2542.44 ± 629.38 vs. 1542.86 ± 502.86; RFcsa (cm^2^): 2.04 ± 0.64 vs. 2.34 ± 0.61; MRC-score: 41.22 ± 3.42 vs. 51.42 ± 2.72, all

**Conclusion:**

The plasma GDF-15 concentration level was significantly associated with skeletal muscle function and muscle wasting on day 7 in ICU patients with mechanical ventilation. Therefore, it can be concluded that the plasma GDF-15 level on the 7th day has a high diagnostic yield for ICU-acquired muscle weakness, and it can predict the 90-day survival status of ICU mechanically ventilated patients.

## 1. Introduction

Acute muscle loss in critically ill patients causes weakness, ranging from mild loss of strength and muscle wasting to profound muscle weakness, weaning difficulties, and quadriplegia [1]. In this context, severe weakness is known as intensive care unit-acquired weakness (ICU-AW) [2]. It is a common clinical problem that is associated with significant morbidity and mortality [3]. Muscle wasting and consequent loss of strength, which result from critical illness, vary both in severity and duration. Overall, in critically ill patients treated with intubation and mechanical ventilation for one week or more, 60% have significant muscle weakness at awakening [4]. Therefore, early diagnosis and timely intervention of ICU-AW is crucial. However, until now there is no “gold standard” for ICU-AW assessment and diagnosis [5], and especially there is a lack of certain biomarkers with high diagnostic value. Growth differentiation factor-15 (GDF-15) is a transforming growth factor *β* (TGF-*β*) protein that has been shown to be involved in the development of muscle atrophy in a variety of diseases, including chronic obstructive pulmonary disease (COPD), cancer, and pulmonary hypertension, and is associated with skeletal muscle wasting [6–8]. The present study is an observational research. Its aim is to clarify the connection between GDF-15 and muscle wasting in ICU patients treated with mechanical ventilation, as well as to evaluate its utility as an early biomarker of muscle loss in the diagnosis of ICU-AW. The ultimate goal is to make clinical interventions more timely and effective.

## 2. Research Objects and Methods

### 2.1. Research Objects

Patients with acute respiratory failure, who received invasive mechanical ventilation in the ICU ward from June 2017 to January 2019 were screened.

#### 2.1.1. Inclusion Criteria

Age ≥18 years; conscious patients; invasive mechanical ventilation treatment; estimated mechanical ventilation treatment for more than 5 days; ICU length of stay ≥7 days.

#### 2.1.2. Exclusion Criteria

Age <18 years; unconscious patients unable to cooperate with the examination; spinal cord injury; acute stroke; lower limb fracture; a history of cognitive dysfunction or neuromuscular disease; receiving muscle relaxant treatment; acute and chronic heart failure; severe edema.

#### 2.1.3. Elimination Criteria

Patients with hospital length of stay less than 7 days due to death or automatic discharge.

### 2.2. Ethics Statement

The research was conducted through the medical research registration system of the National Health Commission of China and the protocol was approved by the ethics committee of the Lianyungang Clinical College of Nanjing Medical University. The approval number was LCYJ20170528001. Before the beginning of the study, written informed consent was obtained from the patient's legal representatives. Patient records/information was anonymized and deidentified prior to analysis.

### 2.3. Research Methods

#### 2.3.1. Quantification of Plasma GDF-15 Levels

Blood samples were collected on day 1, day 4, and day 7. Plasma was separated from blood collected into EDTA tubes by centrifugation at 3500 rpm for 10 minutes, within 2 hours of collection. Samples were stored at −80°C until they were processed. The plasma GDF-15 concentration was determined by enzyme-linked immunosorbent assay (ELISA). The test kit was purchased from Life-tech, USA. Results were analyzed according to the manufacturer's guidelines.

#### 2.3.2. Ultrasound Measurement of RFcsa

To ensure measurement accuracy and reduce technical errors, a unified standard operating procedure was used to measure the rectus femoris cross-sectional area (RFcsa). The patients were positioned supine on the bed with their legs flat and the leg muscles relaxed. The anterior superior iliac spine (ASIS) and the point 60% of the distance from the ASIS to the superior border of the patella were identified. The ultrasound probe was positioned perpendicular to the axis of the leg. The same point was used in the follow-up measurements for each patient [9]. All measurements were taken by two specially trained ultrasound technicians. Each time, each technician obtained three measurements from each patient. In the beginning, in order to determine the coefficient of variation (CV), 2 additional measurements were performed for the first 15 patients on a separate occasion on the same day (3 × 3 measurements for the first 15 patients). The CV for these first 15 patients was controlled within 8.1% and the CV for the other patients was 8.3%, demonstrating good interobserver reliability. Inadequate ultrasound images were defined as those where the edges of the RF muscle could not be adequately defined to calculate the RFcsa. This occurred where the patient was very edematous and in these cases the patient data were excluded.

#### 2.3.3. Muscle Strength Assessment Using the MRC-Score

On day 1, day 4, and day 7, all sedative and analgesic medications were discontinued, keeping the patient conscious and able to effectively cooperate with the physical examination. In all enrolled patients, remifentanil was used for analgesia, and dexmedetomidine and propofol were used for sedation during mechanical ventilation. The Richmond Agitation-Sedation Scale (RASS) was used to assess the depth of sedation. The Medical Research Council (MRC) score for muscle strength assessment was used to evaluate the functions of the six major muscle groups responsible for shoulder abduction, elbow flexion, wrist extension, hip flexion, knee extension, and ankle dorsiflexion, all scored bilaterally, about 1 to 2 hours after discontinuing sedative drugs [10]. More importantly, a valid MRC assessment can only be performed when the RASS score ranges between 0 and 1, and the patient is awake, cooperative, and capable of contracting the extremities with maximal force. The MRC-score grades strength from 0 (no contraction at all) to 5 (normal muscle strength) in the functional muscle groups from each extremity. Individual MRC sum scores obtained from predefined muscle groups can be combined in a sum score that ranges between 0 and 60, which yields a global estimation of motor function, and ICU-AW can be diagnosed when <48 [11]. The strength score was simultaneously determined by two trained researchers. A good interobserver reliability was demonstrated, and the highest score of three assessments was taken. Based on whether the 7th day MRC-score was <48, 50 cases were included in the ICU-AW group and 45 cases in the non-ICU-AW group.

### 2.4. Statistical Processing

Data processing and mapping were performed using the SPSS 22.0 statistical software and GraphPad Prism 6.0. The measurement data were expressed as mean ± standard deviation (*x* ± *s*). Differences between the two groups were compared using two independent sample *t*-tests. The *χ*^2^ test was used to compare the count data, and the difference was statistically significant at *P* < 0.05. Patients were divided into an ICU-AW group and a non-ICU-AW group according to the 7th day MRC-score. The differences in plasma GDF-15 level, MRC-score, and RFcsa between the two groups were compared on the 1st, 4th, and 7th day after being admitted to the ICU. Then, the correlation between plasma GDF-15 level, RFcsa loss, and MRC-score on day 7 was investigated using the Pearson correlation coefficient. The receiver operating characteristic curve (ROC) was used to evaluate the diagnostic sensitivity of the plasma GDF-15 level, RFcsa loss, and % decrease in RFcsa on the 7th day to the diagnosis of ICU-AW in mechanically ventilated patients. Moreover, the predictive value of GDF-15 on the 90-day survival status of patients was assessed by Kaplan–Meier survival curves.

## 3. Results

**Figure 1:**
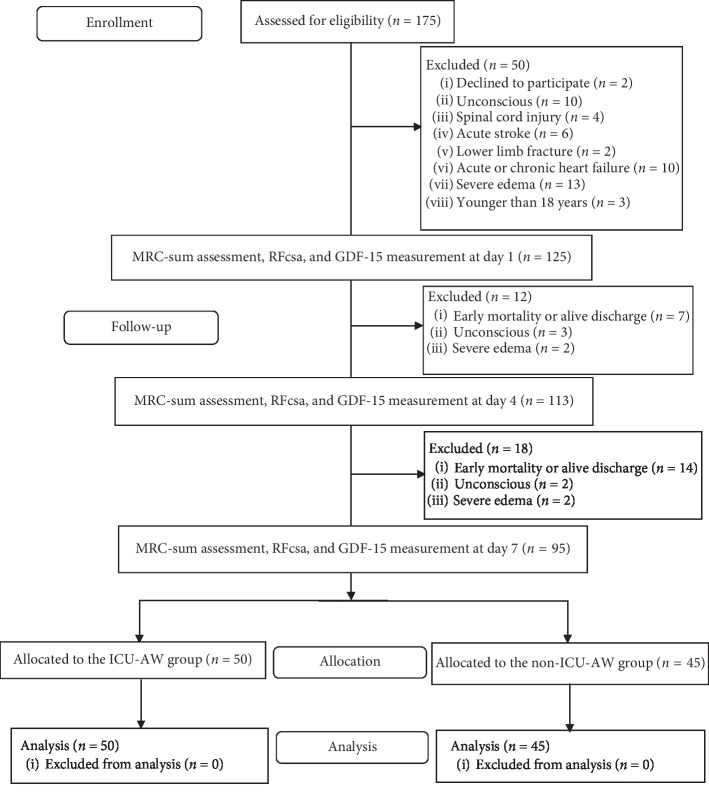
Overview of the included patients.

175 patients were screened at ICU admission (day 1) in this study. 55 cases were excluded because they did not meet the inclusion criteria, including 2 cases who refused to participate in the study, 10 cases who were unconscious, 4 cases of spinal cord injury, 6 cases of acute stroke, 2 cases of lower limb fracture, 10 cases of acute or chronic heart failure, 13 cases with severe edema at ICU admission, and 3 cases who were younger than 18 years. A total of 125 cases were enrolled. There were 21 dropouts due to ICU stay shorter than 7 days, and 4 dropouts due to severe edema and 5 dropouts due to unconsciousness. From the remaining 95 patients, 50 comprised the ICU-AW group and 45 the non-ICU-AW group based on whether the patient's 7th day MRC-score was <48. (Figure 1).

### 3.1. Comparison of Baseline Levels and General Clinical Data

No significant differences (*P* > 0.05) were found in the gender, age, body weight, body mass index (BMI), comorbidities, smoking history, APACHE II, SOFA score, and cause of mechanical ventilation between the ICU-AW group and the non-ICU-AW group. However, statistically significant differences were found in the length of mechanical ventilation, ICU length of stay, and hospital length of stay between the two groups (*P* < 0.05) (Table 1).

### 3.2. Comparison of Plasma GDF-15 Level, MRC-Score, and RFcsa Between the Two Groups

**Figure 2:**
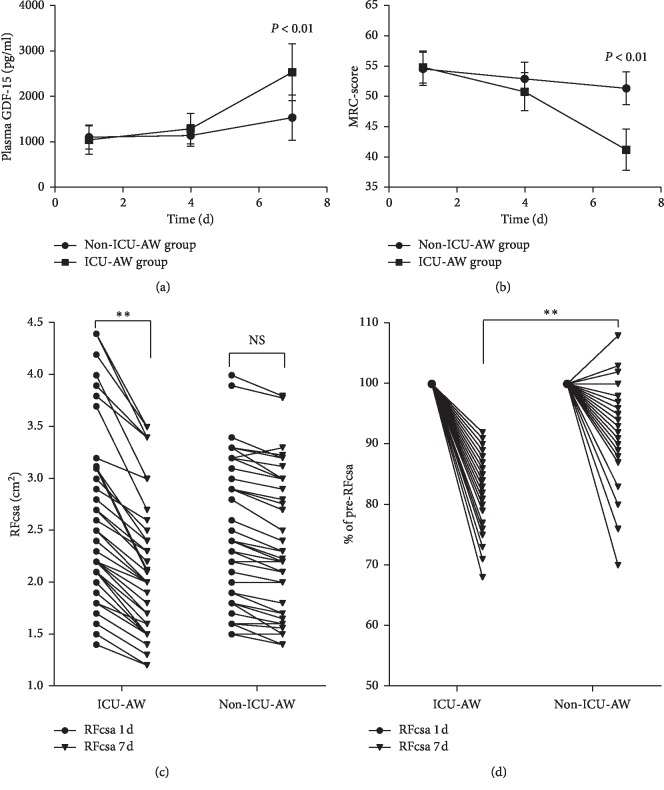
Trends of plasma GDF-15 and MRC-score at different time points in the two groups.

Data were collected on day 1, day 4, and day 7. As the treatment time increased, the plasma GDF-15 level was significantly increased, the ICU-AW group demonstrated a significant decreasing trend in the MRC-score and RFcsa, while no significant changes were found in the non-ICU-AW group (Figures 2(a) and 2(b)). On the 7th day, the RFcsa in the ICU-AW group was significantly reduced compared with that on the 1st day, indicating a significant muscle loss, while the muscle loss in the non-ICU-AW group was not significant (Figures 2(c) and 2(d)). A comparison between the two groups on the 7th day showed that, in the ICU-AW group, the plasma GDF-15 level was significantly higher than that in the non-ICU-AW group, while the RFcsa and the MRC-score were significantly lower than those in the non-ICU-AW group (*P* < 0.05). However, on the 1st day, there was no difference in the plasma GDF-15 levels, MRC-score, and RFcsa between the two groups (*P* < 0.05) (Table 2).

### 3.3. Correlation Analysis of Plasma GDF-15 Level, RFcsa Loss, % Decrease in RFcsa, and MRC-Score on the 7th Day

**Figure 3:**
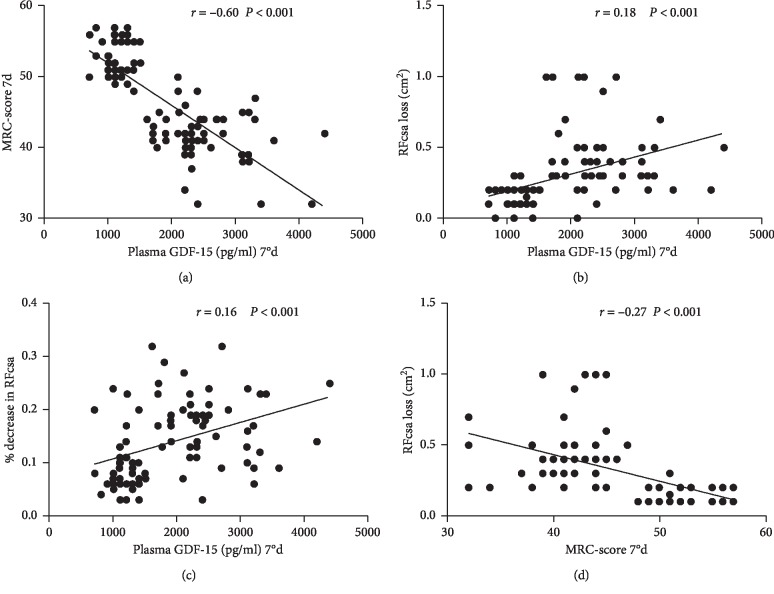
Correlation analysis of GDF-15 level, RFcsa loss, % decrease in RFcsa, and MRC-score on day 7.

The correlation analysis showed that on the 7th day, the plasma GDF-15 level was significantly negatively correlated with the MRC-score (*r* = −0.60) (Figure 3(a)), while it was significantly positively correlated with the RFcsa loss (*r* = 0.18) and the % decrease in RFcsa (*r* = 0.16) (Figures 3(b) and 3(c)). In addition, the RFcsa loss was significantly negatively correlated with the MRC-score (*r* = −0.27) (all *P* < 0.001) (Figure 3(d)).

### 3.4. Diagnostic Value of Plasma GDF-15, RFcsa Loss, and % Decrease in RFcsa for ICU-AW in Mechanically Ventilated Patients

**Figure 4:**
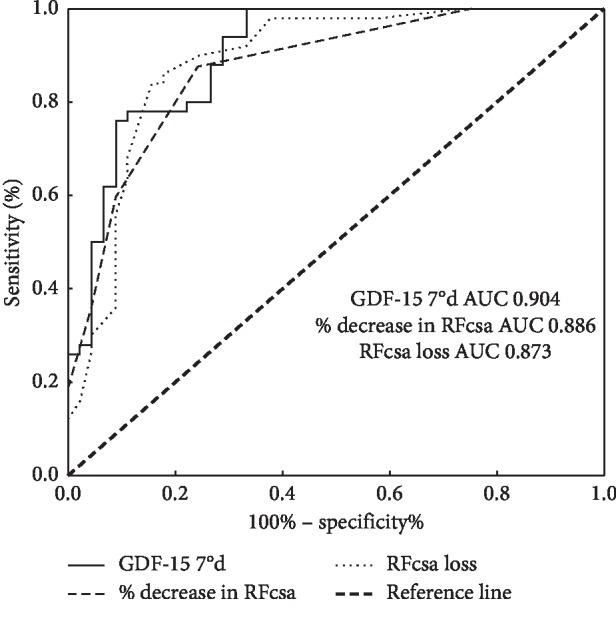
ROC curves of plasma GDF-15, RFcsa loss, and % decrease in RFcsa for ICU-AW diagnosis in mechanically ventilated patients.

The ROC curve analysis demonstrated that the plasma GDF-15 level, RFcsa loss, and % decrease in RFcsa on day 7 had predictive value for ICU-AW diagnosis in mechanically ventilated patients. It was determined that the point on the ROC curve where the Youden index (sensitivity + specificity − 1) was the largest, was the optimal cutoff value. The area under the ROC curve (AUC) of GDF-15 was 0.904 with an optimal cutoff value of 1722 pg/ml, a sensitivity of 94.0%, and a specificity of 71.1%. The AUC of RFcsa loss was 0.873 with an optimal cutoff value of 0.25 cm^2^, a sensitivity of 88.0%, and a specificity of 80.0%. The AUC of the % decrease in RFcsa was 0.886 with an optimal cutoff value of 12.75%, a sensitivity of 84.0%, and a specificity of 84.4% (all *P* < 0.001) (Figure 4).

### 3.5. Effect of Plasma GDF-15 Level on the 90-Day Survival Rate of ICU Patients with Mechanical Ventilation

**Figure 5:**
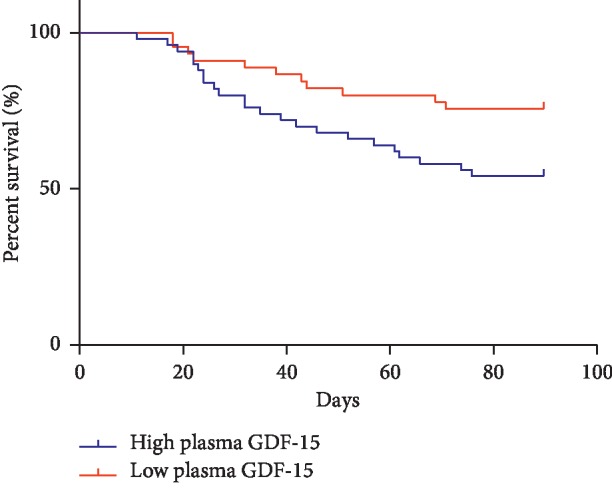
Kaplan–Meier survival curve.

The 90-day survival status of the enrolled patients was assessed, and all patients were divided into a high plasma GDF-15 level group and a low plasma GDF-15 level group based on the optimal cutoff value of GDF-15 on day 7. The 90-day Kaplan–Meier survival curve showed that the 90-day survival rate in the high plasma GDF-15 level group was 54.00%, while that in the low plasma GDF-15 level group was 75.56%. The difference was statistically significant (*P* < 0.05) (Figure 5).

## 4. Discussion

The incidence of ICU-AW in critically ill patients is relatively high. Until today, it is often unlikely to be detected in time due to unconsciousness or the use of sedative drugs, which result in delays in diagnosis and treatment [12]. Therefore, it is very important to find some objective biomarkers for the early diagnosis and evaluation of ICU-AW. In recent years, GDF-15, a protein of the TGF-*β* family, has been shown to be associated with muscle atrophy in some chronic diseases. It has also been proven to be an ideal biomarker for muscle and weight loss in patients with pulmonary hypertension [13]. GDF-15 is gradually receiving more attention in the field of critical care due to its possible involvement in the pathogenesis of ICU-AW [14].

In the present study, it was found that in the ICU-AW group, the length of mechanical ventilation, the ICU length of stay, and the hospital length of stay were significantly longer than in non-ICU-AW group, which was consistent with previous studies [15], This indicates that patients with ICU-AW are prone to respiratory muscle weakness, resulting in prolonged ventilator weaning, which can further complicate the course of critically ill patients and affect their prognosis. As the treatment time increased, the MRC-score of the ICU-AW group gradually decreased, while the plasma GDF-15 level showed a significant increasing trend. Until the 7th day, the GDF-15 level of the ICU-AW group was significantly higher than that of the non-ICU-AW group. At the same time, the bedside ultrasound examination revealed that the cross-sectional area of the patient's left rectus femoris muscle was significantly reduced. These results demonstrated that there was significant acute skeletal muscle wasting in ICU-AW patients [16]. Muscle protein metabolism disorder has been shown to play an important role in the pathogenesis of ICU-AW [17]. As it is known, excessive catabolism is an important metabolic feature in critically ill patients, especially the elderly, and muscle protein breakdown is an important mechanism of catabolism, which can directly promote the occurrence of ICU-AW [18]. It is currently believed that muscle protein degradation is mainly carried out by the ubiquitin-proteasome and autophagy-lysosome pathways. When the protein degradation pathway is abnormally activated, protein degradation accelerates the reduction of muscle mass, leading to muscle atrophy [19]. The cytokine GDF-15 is one of the important regulators of the protein synthesis/catabolism balance, which may be involved in the activation process of the above proteolytic pathways [20]. When it is abnormally expressed in the human body, it can reduce muscle protein synthesis and cause muscle atrophy. A study by Bloch et al. [21] found that GDF-15 was elevated in high-risk patients after cardiac surgery in the ICU. Subsequently, they conducted some *in vitro* studies to confirm that GDF-15 can cause myotube atrophy. In addition, their study demonstrated that GDF-15 may inhibit muscle miRNA expression to promote muscle atrophy by increasing the sensitivity of TGF-*β* signaling. This conclusion resulted from their observations on muscle biopsies of the rectus femoris muscle in patients with ICU-AW.

At present, in clinical practice, the consensus on the diagnosis of ICU-AW is still the evaluation of the six major muscle groups by the applied MRC muscle strength score, with a total score of <48 as the diagnostic criterion for ICU-AW [15]. However, the disturbance of consciousness and ICU sedation treatment severely limit the use of the MRC-score. Therefore, the patients who were enrolled in the present study should be conscious enough in order to be able to cooperate with the physical examination. This also proved that more methods in diagnosing ICU-AW are urgently needed to be explored. Bedside ultrasound has a huge advantage in assessing muscle mass due to its convenience, noninvasiveness, and repeatability [22–24], and it has important guiding significance for the treatment of critically ill patients. However, due to that it has not been proven to be related to muscle function, therefore it is not widely used in the diagnosis of ICU-AW. In this study, it was found that on the 7th day, the plasma GDF-15 level was significantly negatively correlated with the MRC-score (*r* = −0.60), while it was significantly positively correlated with the RFcsa loss (*r* = 0.18) and the % decrease in RFcsa (*r* = 0.16). Moreover, the RFcsa loss was significantly negatively correlated with the MRC-score (*r* = −0.27). The plasma GDF-15 level, RFcsa loss, and % decrease in RFcsa on day 7 were found to have predictive value for ICU-AW diagnosis in mechanically ventilated patients. All these results demonstrated that GDF-15 is a good biomarker for muscle loss and has a good correlation with bedside ultrasound monitoring of rectus femoris muscle mass loss. They can synergistically assess the degree of muscle loss in ICU-AW patients. Furthermore, they have certain correlations with the MRC-score, which reflects the patient's muscle function. Consequently, they can complement each other and work together. Especially in patients with coma or receiving sedation, where the MRC muscle strength score cannot be assessed, the plasma GDF-15 level and the RFcsa loss may diagnose and determine whether patients have ICU-AW at an early stage.

At the same time, this study found that a higher plasma GDF-15 level on the 7th day may suggest a lower 90-day survival rate for ICU mechanically ventilated patients, which was consistent with an increase in mortality among critically ill patients who were diagnosed with ICU-AW [18]. This also indicated that ICU-AW severely reduces the quality of life of critically ill patients and affects their prognosis. It was discussed that this may be due to the weakness of the respiratory muscles of ICU-AW patients, which prolongs the ventilation time, and as a result, increases the incidence of complications, such as ventilator-associated pneumonia and the failure rate of extubation of tracheal intubation [25]. In addition, the weakness of the limbs increases the bed-rest time of the patient, leading to bedridden complications [10].

## 5. Limitations

This study was an observational research. It can be only suggested that GDF-15 is associated with muscle loss in patients with ICU-AW; however, it did not further prove the causal relationship between GDF-15 and muscle loss. Moreover, in this study, the mechanism of muscle atrophy in ICU-AW patients was not further explored. In addition, the study was a single-center trial with a small number of cases, and further multicenter and large-sample clinical studies are needed to confirm the value of GDF-15 in the diagnosis and evaluation of ICU-AW.

## 6. Conclusions

The sustained elevation of plasma GDF-15 concentration level has been found significantly associated with skeletal muscle function and muscle wasting in ICU patients with mechanical ventilation. The plasma GDF-15 level on the 7th day has a high diagnostic yield for ICU-acquired muscle weakness, and it has been shown to be an “ideal” candidate biomarker for muscle mass loss in ICU-AW patients. A persistently elevated plasma GDF-15 concentration suggests that patients may have a poor prognosis, and the plasma GDF-15 level on the 7th day can predict the 90-day survival status of ICU mechanically ventilated patients.

## Figures and Tables

**Table 1:** Comparison of baseline levels and clinical features between the two groups.

	ICU-AW group (*n* = 50)	Non-ICU-AW group (*n* = 45)	*t*/*χ*^2^	*P* value
Baseline characteristics				
Age (years)	58.75 ± 13.58	60.32 ± 15.63	0.524	0.602
Male/total	32/50	29/45	0.045	0.964
Weight (kg)	61.43 ± 8.93	59.57 ± 11.68	0.877	0.383
BMI (kg/m^2^)	23.79 ± 3.41	24.47 ± 4.54	0.831	0.408
SOFA	8.23 ± 3.18	7.21 ± 3.67	1.451	0.150
APACHE II	16.35 ± 7.21	15.79 ± 8.12	0.356	0.723
Smoking history	18/50	15/45	0.001	0.983
Comorbidities	22/50	18/45	0.003	0.956
Cause of MV				
Pneumonia	12/50	13/45	0.540	0.589
AECOPD	19/50	17/45	0.022	0.982
Pulmonary contusion	7/50	6/45	0.094	0.925
** **ARDS	6/50	4/45	0.493	0.622
Others	6/50	5/45	0.135	0.893
Length of MV (h)	196.68 ± 51.36	167.52 ± 78.72	2.159	0.033
ICU length of stay (days)	11.25 ± 3.37	9.76 ± 2.31	2.486	0.015
Hospital length of stay (days)	20.54 ± 6.37	17.76 ± 4.93	2.359	0.020

Data are mean ± standard deviation or number/total. Comorbidities include hypertension, diabetes, chronic kidney disease, cirrhosis, and history of trauma surgery. BMI: body mass index, SOFA: sequential organ failure assessment, APACHEII :  Acute Physiology and Chronic Health Evaluation II, MV: mechanical ventilation, AECOPD: acute exacerbation of chronic obstructive pulmonary disease, ARDS: acute respiratory distress syndrome.

**Table 2:** Comparison of plasma GDF-15 level, MRC-score, and RFcsa index between the two groups.

	Groups	Day 1	Day 4	Day 7
GDF-15 (pg/ml)	ICU-AW group (*n* = 50)	1047.16 ± 314.43	1295.44 ± 338.15	2545.44 ± 629.38
	Non-ICU-AW group (*n* = 45)	1109.22 ± 268.62	1146.35 ± 239.22	1542.86 ± 502.86
	*t*	1.03	2.46	8.52
	*P* value	0.31	0.16	0.00
RFcsa (cm^2^)	ICU-AW group (*n* = 50)	2.49 ± 0.78	2.27 ± 0.52	2.04 ± 0.64
	Non-ICU-AW group (*n* = 45)	2.51 ± 0.66	2.38 ± 0.65	2.34 ± 0.61
	*t*	0.096	1.30	2.33
	*P* value	0.92	0.19	0.02
MRC-score	ICU-AW group (*n* = 50)	54.92 ± 2.65	50.84 ± 3.14	41.22 ± 3.42
	Non-ICU-AW group (*n* = 45)	54.64 ± 2.74	52.97 ± 2.76	51.42 ± 2.72
	*t*	0.49	3.51	17.51
	*P* value	0.62	0.01	0.00

## Data Availability

The data used to support the findings of this study are available from the corresponding author upon request.
